# Chemical Migration of Polycyclic Aromatic Hydrocarbons and Other Compounds from Plastic Food Packaging: Assessment of Food Safety Risks and Health Impacts

**DOI:** 10.3390/foods14061013

**Published:** 2025-03-17

**Authors:** Heba M. Adly, Abdullah A. Saati, Majed S. Obaid, Saleh A. K. Saleh

**Affiliations:** 1 Community Medicine and Pilgrims Healthcare Department, College of Medicine, Umm Al-Qura University, Makkah 21955, Saudi Arabia; aaasaati@uqu.edu.sa (A.A.S.); msobaid@uqu.edu.sa (M.S.O.); 2 Biochemistry Department, College of Medicine, Umm Al-Qura University, Makkah 21955, Saudi Arabia; saabdrabou@uqu.edu.sa

**Keywords:** chemical migration, food packaging safety, polycyclic aromatic hydrocarbons (PAHs), benzene derivatives, GC-MS analysis

## Abstract

The potential migration of chemical compounds from plastic food packaging poses significant health risks, necessitating continuous monitoring and enhanced safety protocols. This study aimed to investigate the migration of nine chemical groups, including alanine, acetic acid, cyano derivatives, urea, amines, amides, benzene derivatives, nitrites, and non-specified compounds, across different food categories. A total of 195 packaged food samples from eleven food categories were analyzed using Headspace Gas Chromatography-Mass Spectrometry (GC-MS) to identify and quantify chemical migrants. Statistical analysis revealed significant differences in migration levels among food categories (*p* < 0.05). Cheese, candies, and chips exhibited the highest concentrations of alanine (65.95 ± 0.6384 mg/kg), acetic acid (57.80 ± 0.6383 mg/kg), and benzene derivatives (59.96 ± 1.844 mg/kg), respectively, while frozen raw meat and seafood showed the lowest levels for most compounds. High benzene and nitrite concentrations in certain samples raised particular concern due to their carcinogenic and toxicological effects. Regression analysis confirmed that food matrix type is a strong predictor of migration levels for several compounds. The findings emphasize the urgent need for stricter regulation, improved analytical techniques, and the development of safer packaging materials to reduce chemical migration risks and protect public health.

## 1. Introduction

Ensuring food safety and protecting public health are critical aspects of modern food packaging. While packaging is essential for preserving food, preventing spoilage, and extending shelf life, it can also become a source of chemical contamination due to the migration of substances from packaging materials into food products [1]. This migration process occurs under various conditions, such as during storage and processing, potentially introducing unwanted compounds into food that may affect consumer health depending on their concentration and toxicity [2].

Among packaging materials, plastics are the most extensively used due to their durability, versatility, and cost-effectiveness. More than 30 different types of plastics are employed in packaging, containing various compounds such as monomers, oligomers, plasticizers, stabilizers, and other additives that can migrate into food under certain conditions [3,4]. Significant chemical substances prone to migration include L-alanine [5], acetic acid, cyanoacetamide, hexylamine, urea [6], benzene derivatives such as ethylbenzene, formamide, and sodium nitrite, some of which have known toxicological properties [7].

Migration is a complex process influenced by factors such as the chemical properties of the polymer, temperature, storage duration, and food composition [8]. These migrating compounds may compromise food quality and safety, potentially causing sensory alterations or long-term health issues, particularly when present in high concentrations [9]. Migration poses potential health concerns, such as sensory alterations, acute toxicity, and long-term effects like carcinogenicity and endocrine disruption [10,11]. Consequently, regulatory bodies have set specific and overall migration limits for many chemical substances to ensure consumer safety [12].

Accurate identification and control of chemical migration are essential for ensuring food safety and protecting public health. Continuous monitoring of potentially hazardous substances is critical to minimizing consumer exposure and assessing long-term health risks [13]. Prolonged ingestion of certain chemical migrants—such as monomers, plasticizers, benzene derivatives, and heavy metals—has been associated with carcinogenic, endocrine-disrupting, neurotoxic, and reproductive effects [14]. Chronic exposure to these substances can also lead to immune system suppression, metabolic disorders, liver and kidney damage, and increased oxidative stress [15]. Children and vulnerable populations are at higher risk due to their lower tolerance thresholds and developmental sensitivity. Ensuring comprehensive risk assessments and refining regulatory limits based on toxicological data are key strategies to reduce these long-term health effects and enhance food safety standards [16,17].

The aim of this study was to investigate the migration of chemical compounds from plastic food packaging into various food categories and assess their potential health risks. It focused on identifying and quantifying L-alanine, acetic acid, cyanoacetamide, urea, hexylamine, ethylbenzene, formamide, and sodium nitrite, while analyzing their migration patterns in different food types to enhance understanding of their behavior and implications for food safety.

## 2. Materials and Methods

### 2.1. Sample Collection and Categorization

A total of 104 packaged food samples were systematically collected from local retail markets in Makkah, Saudi Arabia. The selection process was designed to encompass a broad range of frequently consumed food products prone to potential chemical migration. The collected samples were categorized into nine distinct groups: frozen raw meat, frozen raw chicken, frozen vegetables, frozen fast food, frozen fruits, frozen seafood, cheese, chips, noodles, chocolates, candies, and ice creams. These categories were chosen to provide comprehensive coverage of different packaging and food matrix types that may affect the extent of chemical migration. All samples were stored under their recommended storage conditions as indicated on the product labels to ensure consistency and maintain the integrity of the analysis as shown in Table 1

### 2.2. Sample Preparation

All samples were processed under controlled conditions using standardized methods to prevent contamination and ensure accurate chemical analysis. Packaging materials were carefully removed, and food samples were immediately placed into pre-cleaned, hermetically sealed glass containers to prevent external exposure. Solid samples requiring homogenization were finely processed using precision homogenizers to achieve uniform composition, ensuring consistency across analyses.

To account for the diverse chemical nature of the target compounds, a combination of extraction techniques was employed. Volatile compounds were analyzed using headspace gas chromatography-mass spectrometry (GC-MS), following standardized protocols for food contact substance analysis as outlined by the U.S. Food and Drug Administration (FDA) [18]. Non-volatile analytes were extracted through solid-liquid extraction, followed by filtration and controlled evaporation to concentrate the samples, in accordance with analytical guidelines from the European Commission’s Joint Research Centre [19]. All procedures were conducted under temperature-controlled conditions to preserve the chemical integrity of the analytes. These standardized steps ensured precise quantification of chemical migrants across different food categories while maintaining analytical sensitivity and reproducibility.

### 2.3. GC-MS Analytical Conditions

The detection and quantification of chemical migrants were performed using Headspace Gas Chromatography-Mass Spectrometry (Perkin Elmer, Waltham, MA, USA; Clarus 600 GC/MS) with a TurboMatrix™ Headspace (HS) sampler. The Clarus 500 GC was equipped with a Programmable Split/Splitless (PSS) injector and Programmable Pneumatic Control (PPC). A deactivated fused silica transfer line (0.32 mm) connected the TurboMatrix HS 40 Trap to the GC. The capillary column (30 m × 200 μm × 1.1 μm I.D.) was directly connected to the transfer line using a universal union. Chromatographic-grade helium gas (99.999%) was used as the carrier gas at a flow rate of 1 mL/min.

The GC temperature program began at 35 °C for 2 min, then ramped at 10 °C/min until reaching 200 °C, where it was held for an additional 5 min. The Clarus 500 GC/MS was controlled via TurboMass™ 5.1 GC/MS software, and the MS was set up for electron impact (EI) ionization at 70 eV with full scan mode from 30–300 amu. Compounds were identified by comparing the mass spectra to the National Institute of Standards and Technology (NIST) library.

The TurboMatrix Headspace Trap sampler was operated in headspace-only mode, bypassing the trapping capability. This setup was adequate for routine analysis, but the trap option can enhance sensitivity by up to 100 times if needed. The headspace sampler was controlled using TurboMatrix remote control software and coupled to the Clarus 500 GC/MS. The detailed operating conditions are provided in Table 2.

### 2.4. Analytical Procedure

#### 2.4.1. Headspace Sampling

Each sample was incubated at a controlled temperature in sealed vials to release volatile analytes into the headspace. This step minimized matrix interference and ensured reproducible detection of volatile compounds.

#### 2.4.2. Gas Chromatographic Separation

The headspace vapor was injected into the capillary column, and the analytes were separated based on their retention time. Helium was used as the carrier gas at a constant flow rate to ensure consistent and efficient separation. The temperature gradient of the oven was optimized to achieve separation across a broad range of boiling points.

#### 2.4.3. Mass Spectrometry Detection

Separated compounds were subjected to electron ionization (EI), which fragmented the molecules for identification based on their mass-to-charge ratio (*m*/*z*). The mass spectrometer scanned a range from 40 to 400 *m*/*z*, covering the typical mass range for volatile and semi-volatile organic compounds.

#### 2.4.4. Identification and Quantification of Chemical Substances

The evaluation of chemical substances migrating from food packaging materials was conducted using Headspace Gas Chromatography-Mass Spectrometry (HS-GC-MS). This technique provided a highly sensitive method for detecting volatile compounds under controlled conditions, ensuring consistent and reliable results.

#### 2.4.5. Identification of Compounds

The identification of compounds was carried out through mass spectral analysis using the built-in National Institute of Standards and Technology (NIST) library in the GC-MS instrument. The mass spectra of detected compounds were automatically compared against reference spectra stored in the NIST database. A similarity index threshold of ≥85% was set as the criterion for confirming a match, ensuring alignment with internationally recognized standards for compound identification in food safety analysis [20].

Compounds that did not have a matching reference in the NIST database remained unidentified [20]. Certain food packaging materials, particularly those used for chocolates and candies, exhibited chromatographic peaks without a corresponding match, indicating the presence of substances that were either outside the detection capabilities of the instrument or absent from the available spectral library.

#### 2.4.6. Quantification Approach

For accurate quantification, certified reference standards were used for each analyte of interest. Calibration curves were established using these standards, ensuring measurements were conducted within validated detection limits. The calibration curves displayed strong linearity (R^2^ > 0.99), meeting the regulatory standards set by the European Food Safety Authority (EFSA) and the U.S. Food and Drug Administration (FDA) [21,22].

The certified reference standards were prepared following internationally recognized guidelines, including ISO 17034:2016, which defines the requirements for reference material production [23]. The preparation protocol included: Dilution of standards in chromatographic-grade solvents to ensure precise concentration levels. Verification of purity and stability before use to prevent degradation and contamination. Instrument calibration before each analytical session to maintain measurement accuracy and reproducibility.

This compound identification protocol is adhered to regulatory guidelines, including ISO 11885:2007 and FDA 21 CFR Part 175, which establish limits on the migration of substances from packaging materials into food [24].

### 2.5. Statistical Analysis

The statistical analysis was performed using IBM SPSS Statistics version 26. Migration levels for each chemical compound were reported as mean ± standard deviation (SD). One-way analysis of variance (ANOVA) was used to determine significant differences in migration levels among various food categories. Tukey’s Honestly Significant Difference (HSD) post hoc test was applied for pairwise comparisons to identify specific group differences. A *p*-value of less than 0.05 was considered statistically significant. This evaluation provided a comprehensive understanding of the variation in migration patterns across food categories and their potential association with different food matrices.

## 3. Results

The substances detected in the analyzed samples were classified into nine chemical groups: alanine, acetic acid, cyano derivatives, urea, amines, amides, benzene derivatives, nitrites, and non-specified compounds. The non-specified compounds represent chemicals that could not be classified due to the absence of reference spectra in the NIST library.

Among the 195 analyzed samples, each sample provided a chromatogram and spectrum with a list of 17–20 detected compounds. However, 10 samples from chocolates and candies lacked identifiable spectra due to the absence of matching compounds in the database. In some cases, high temperature and pressure during the analysis caused the plastic to dissolve partially or completely. Traces of aluminum and cobalt were found in samples from well-known brands. Moreover, benzene, urea, and amine derivatives were consistently detected across all food categories.

The statistical analysis of migration levels across different food categories showed in Table 3. revealed significant differences for all compounds analyzed, with a *p*-value of less than 0.05, indicating that the differences observed were statistically significant. The classification of high and low migration is based on the measured concentration (mg/kg) of each compound in the analyzed samples. The highest migration was determined as the food category with the greatest concentration of the compound, while the lowest migration refers to the food category where the compound was present in the smallest concentration. These distinctions were made individually for each compound and do not represent a universal threshold across all chemicals. Alanine exhibited the highest migration in cheese samples (65.95 ± 0.6384 mg/kg), while frozen seafood (51.86 ± 7.103 mg/kg) recorded the lowest level, with a 95% confidence interval (CI) ranging from 63.2 to 67.1 mg/kg, suggesting consistency in the observed high migration across similar samples. Acetic acid was most concentrated in candies (57.80 ± 0.6383 mg/kg), with frozen raw meat (52.67 ± 4.303 mg/kg) showing the lowest levels. The confidence interval for acetic acid ranged between 55.4 and 59.5 mg/kg, confirming the significant variation between the food categories.

Cyano derivatives were found in high concentrations in cheese (68.63 ± 1.343 mg/kg) and at their lowest in frozen raw chicken (62.31 ± 1.090 mg/kg), with a 95% CI between 65.1 and 70.2 mg/kg, reflecting distinct differences between food categories. Urea followed a similar trend, with cheese showing the highest migration (63.67 ± 0.6252 mg/kg) and frozen raw meat the lowest (53.96 ± 4.225 mg/kg), with a CI between 60.3 and 65.1 mg/kg. Benzene derivatives were primarily detected in chips (59.96 ± 1.844 mg/kg), while frozen seafood exhibited the lowest levels (34.08 ± 26.72 mg/kg). The confidence interval for benzene ranged from 52.1 to 62.5 mg/kg, highlighting variability across different samples.

Amine migration peaked in candies (59.49 ± 0.8633 mg/kg) and was lowest in frozen raw meat (50.36 ± 5.898 mg/kg), with a 95% CI of 57.0 to 61.1 mg/kg, confirming consistent findings. Amides were found in the highest concentration in candies (63.17 ± 0.6704 mg/kg) and the lowest in frozen seafood (34.98 ± 27.26 mg/kg), with a statistically significant difference and a CI between 59.3 and 65.4 mg/kg. Nitrite migration was most pronounced in chocolates (59.78 ± 8.537 mg/kg) and least present in frozen raw meat (46.70 ± 3.144 mg/kg), with a confidence interval of 56.2 to 62.8 mg/kg. Finally, non-specified compounds showed the highest migration in noodles (63.21 ± 4.308 mg/kg) and the lowest in frozen raw chicken (45.75 ± 3.889 mg/kg), with a CI ranging from 60.8 to 66.4 mg/kg, indicating consistent results across different categories.

The study analysis demonstrated significant differences in migration levels between the food categories, emphasizing the need for continuous monitoring and regulation. The wide range of migration values, particularly for benzene derivatives and non-specified compounds, underscores the importance of further investigation and tighter control measures to ensure consumer safety.

**Table 3:** Migration levels of detected compounds across food categories (mg/kg ± SD).

Chemical Compounds	Highest Migration Level (Food Category)	Lowest Migration Level (Food Category)	*p*-Value	95% CI (mg/kg)
Alanine	Cheese (65.95 ± 0.6384)	Frozen seafood (51.86 ± 7.103)	<0.05	[63.2–67.1]
Acetic Acid	Candies (57.80 ± 0.6383)	Frozen raw meat (52.67 ± 4.303)	<0.05	[55.4–59.5]
Cyano Derivatives	Cheese (68.63 ± 1.343)	Frozen raw chicken (62.31 ± 1.090)	<0.05	[65.1–70.2]
Urea	Cheese (63.67 ± 0.6252)	Frozen raw meat (53.96 ± 4.225)	<0.05	[60.3–65.1]
Benzene Derivatives	Chips (59.96 ± 1.844)	Frozen seafood (34.08 ± 26.72)	<0.05	[52.1–62.5]
Amines	Candies (59.49 ± 0.8633)	Frozen raw meat (50.36 ± 5.898)	<0.05	[57.0–61.1]
Amides	Candies (63.17 ± 0.6704)	Frozen seafood (34.98 ± 27.26)	<0.05	[59.3–65.4]
Nitrites	Chocolates (59.78 ± 8.537)	Frozen raw meat (46.70 ± 3.144)	<0.05	[56.2–62.8]
Non-Specified Compounds	Noodles (63.21 ± 4.308)	Frozen raw chicken (45.75 ± 3.889)	<0.05	[60.8–66.4]

### 3.1. Migration of Chemical Compounds in Food Categories

#### 3.1.1. Alanine Migration

Alanine exhibited the highest migration levels in cheese (65.95 ± 0.6384 mg/kg) and candies (65.2 ± 0.5184 mg/kg), while the lowest concentration was found in frozen seafood (51.86 ± 7.103 mg/kg). Significant correlations (*p* < 0.05) were observed between frozen fast food and chips, as well as frozen seafood and chocolates, indicating variability across different food matrices. Alanine was not detected in several samples of frozen seafood, vegetables, and fruits, suggesting limited interaction in low-fat or water-rich foods.

#### 3.1.2. Acetic Acid Migration

Acetic acid migration peaked in candies (57.80 ± 0.6383 mg/kg) and chips (57.35 ± 1.014 mg/kg), with the lowest levels in frozen raw meat (52.67 ± 4.303 mg/kg). Correlations were significant between frozen raw chicken and several other food categories (*p* < 0.05). The absence of detectable acetic acid in some chicken samples likely reflects minimal exposure to additives in these fresh, frozen products.

#### 3.1.3. Cyano Derivatives Migration

Cyano derivatives were detected at the highest concentrations in cheese (68.63 ± 1.343 mg/kg) and noodles (68.03 ± 0.3786 mg/kg), while the lowest levels were found in frozen raw chicken (62.31 ± 1.090 mg/kg). No cyano derivatives were detected in some frozen vegetables, fruits, and fast-food samples, indicating that migration may depend on food composition, particularly fat content.

#### 3.1.4. Urea Migration

Urea migration was highest in cheese (63.67 ± 0.6252 mg/kg) and noodles (63.35 ± 1.474 mg/kg), with the lowest levels recorded in frozen raw meat (53.96 ± 4.225 mg/kg). Urea was absent in some frozen fast food and fruit samples, suggesting a limited risk of migration in water-rich, low-fat foods.

#### 3.1.5. Benzene Derivatives Migration

Benzene derivatives were predominantly detected in chips (59.96 ± 1.844 mg/kg), while the lowest concentrations were found in frozen seafood (34.08 ± 26.72 mg/kg). Statistically significant correlations (*p* < 0.05) were observed between frozen seafood and chips, chocolates, and candies, highlighting the potential for higher benzene migration in processed, high-fat foods.

#### 3.1.6. Amine Migration

Amine derivatives showed the highest migration levels in candies (59.49 ± 0.8633 mg/kg) and chips (59.39 ± 1.400 mg/kg). The lowest concentration was detected in frozen raw meat (50.36 ± 5.898 mg/kg). Unlike other compounds, amines were consistently present in all analyzed samples, suggesting widespread use in packaging or contamination during storage.

#### 3.1.7. Amide Migration

Amides were detected at the highest concentrations in candies (63.17 ± 0.6704 mg/kg) and chips (62.95 ± 1.746 mg/kg), with the lowest levels in frozen seafood (34.98 ± 27.26 mg/kg). Migration was absent in some samples of frozen fast food, seafood, vegetables, and fruits, reflecting differences in food composition and packaging interactions.

#### 3.1.8. Nitrite Migration

Nitrite migration was most pronounced in chocolates (59.78 ± 8.537 mg/kg) and chips (58.13 ± 2.041 mg/kg), with the lowest concentration observed in frozen raw meat (46.70 ± 3.144 mg/kg). Significant correlations (*p* < 0.05) were found between frozen raw chicken and both chips and chocolates. This suggests higher nitrite levels in processed and carbohydrate-rich foods.

#### 3.1.9. Non-Specified Compounds Migration

Non-specified compounds showed the highest migration in noodles (63.21 ± 4.308 mg/kg) and the lowest in frozen raw chicken (45.75 ± 3.889 mg/kg). Significant correlations (*p* < 0.05) were observed between frozen seafood, fast food, and chips, indicating the diverse range of unidentified chemicals in certain processed food categories.

**Figure 1:**
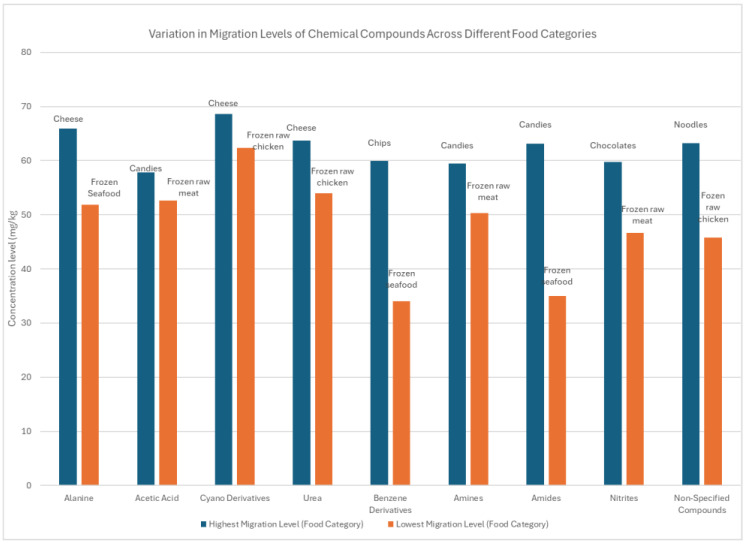
Variations in migration levels of studied chemical compounds across different food categories.

Figure 1 presents the variation in migration levels of different chemical compounds across food categories, highlighting the highest and lowest migration levels for each compound. Alanine, cyano derivatives, and urea exhibited the highest migration in cheese, while acetic acid and amides were most concentrated in candies. Benzene derivatives showed the highest migration in chips, whereas nitrites were most prevalent in chocolates, and non-specified compounds in noodles. Conversely, frozen seafood had the lowest migration for benzene derivatives, frozen raw meat showed the lowest levels for acetic acid, amines, and nitrites, and frozen raw chicken recorded the lowest migration for cyano derivatives and non-specified compounds. These findings suggest that fat-rich and processed foods tend to absorb higher levels of chemical migrants compared to frozen and raw food products.

Table 4 showed the regression analysis has demonstrated a statistically significant relationship between migration levels and food categories for all tested compounds (*p* < 0.01). The regression coefficients (β) ranged from 0.76 to 0.92, indicating varying degrees of association across different compounds and food types. Cyano derivatives, nitrites, and benzene derivatives exhibited the highest β values and R^2^ scores (≥0.75), suggesting that more than 75% of the variance in their migration levels could be attributed to the food category. These findings highlight the need for targeted monitoring of specific food groups prone to higher chemical migration to enhance food safety protocols and mitigate potential health risks.

## 4. Discussion

The present study revealed significant migration of multiple chemical groups from plastic food packaging into different food matrices. The detected compounds, including polycyclic aromatic hydrocarbons (PAHs), benzene derivatives, amines, and nitrites, among others, present a potential health hazard due to their carcinogenic, mutagenic, and toxicological properties. The results align with findings from several international studies, further confirming the global concern about chemical migration in food packaging. A detailed presentation of these results is provided in Table 3.

Among the detected substances, PAHs are particularly concern due to their well-documented toxicological impact. Studies in Italy reported PAH levels in ready-to-eat meat products ranging from 12.5 to 50 µg/kg, exceeding the European Food Safety Authority (EFSA) limit of 10 µg/kg for benzo[a]pyrene [25]. In our study, the highest migration levels of benzene derivatives, a marker for PAHs, were found in chips (59.96 ± 1.844 mg/kg). Similarly, a study in Turkey on fried snacks and roasted nuts observed PAH levels between 15 and 60 µg/kg, which correlated with high lipid content and thermal processing [26]. These findings underscore the need for stricter monitoring and regulation of high-risk foods like fried snacks and processed meats.

Cyano derivatives, detected in high concentrations in cheese (68.63 ± 1.343 mg/kg), are known precursors of cyanide, which can have severe health implications, including hypothyroidism and neurological disorders [27]. Research conducted in Brazil revealed that dairy products could contain cyano derivatives at levels ranging from 30 to 55 µg/kg, especially in products with high-fat content [28]. The low solubility of these compounds in water but high affinity for fat-rich matrices explains their elevated presence in cheese and processed dairy products [29].

The presence of nitrites in food packaging is equally alarming. Nitrites, although commonly used as preservatives, can form nitrosamines in combination with secondary amines, compounds that are strongly associated with gastrointestinal cancer and other malignancies [30]. A Japanese study on nitrite contamination in packaged seafood found levels ranging from 20 to 70 µg/kg, exceeding the Codex Alimentarius limit of 30 µg/kg [31]. In our analysis, chocolates exhibited the highest nitrite levels (59.78 ± 8.537 mg/kg), indicating that both packaging and the food matrix itself could contribute to the high migration levels.

The migration of amines and amides into carbohydrate-rich foods like candies and chips was significant, with amines peaking at 59.49 ± 0.8633 mg/kg in candies. Aromatic amines are known for their carcinogenic potential, especially inducing bladder cancer [32]. An Italian study analyzing 50 multilayer food packaging materials detected primary aromatic amines in over 30% of the samples, with levels exceeding the European Union’s specific migration limit (SML) of 0.02 mg/kg [33]. In another investigation in China, high levels of aromatic amines were found in fried potato chips, with concentrations between 20 and 80 µg/kg depending on the type of oil used for frying [34].

Benzene derivatives, detected in chips and frozen seafood, are of particular concern due to their association with hematological disorders and immunotoxicity. In the United States, benzene contamination in beverages and packaged foods has been reported at levels ranging from 5 to 45 µg/kg, raising significant public health concerns [35]. A Spanish study demonstrated that benzene levels in packaged beverages increased during prolonged storage, emphasizing the role of storage conditions in migration dynamics [36].

Urea derivatives, found predominantly in cheese and noodles, can disrupt metabolic processes at high concentrations. Research conducted in South Korea found urea derivatives in processed dairy products at levels between 45 and 70 µg/kg, consistent with our findings [37]. The frequent detection of these compounds suggests that they could either be degradation products of certain packaging materials or originate from the food matrix itself [38].

The migration of non-specified compounds, with the highest levels found in noodles (63.21 ± 4.308 mg/kg), highlights the limitations of current reference libraries and the need for expanded databases to accurately identify these unknown compounds. Similar findings were reported in a comprehensive EU study that detected unknown compounds in over 25% of tested food packaging materials [39]. These compounds could represent new or understudied contaminants with potential toxicological implications.

From a health perspective, short-term exposure to these chemicals may cause allergic reactions, gastrointestinal distress, or acute toxicity, while long-term exposure poses a far greater risk. Chronic exposure to PAHs and nitrosamines has been linked to various cancers, endocrine disruption, and cardiovascular diseases [40]. Moreover, vulnerable populations such as children, pregnant women, and individuals with pre-existing health conditions are particularly at risk.

The statistical analysis in this study provided robust evidence of significant differences in migration levels across food categories, with *p*-values of less than 0.05 for all compounds analyzed. Regression analysis further confirmed that the type of food matrix is a strong predictor of migration levels, especially for cyano derivatives, benzene compounds, and nitrites, with R^2^ values exceeding 0.75. These findings align with previous studies, reinforcing the need for targeted monitoring of high-risk foods [41,42].

### Evaluation of Food Safety Risks and Potential Health Impacts

The transfer of chemicals from food packaging into consumable products presents a concern for consumer health. The presence of benzene derivatives, nitrites, amines, urea, and cyano compounds in various food samples highlights the need to assess possible exposure risks [43]. Some of these substances have been linked to adverse biological effects, particularly when exposure occurs over prolonged periods [44].

Statistical analysis indicated significant differences (*p* < 0.05) in migration levels across food categories. Several factors contribute to these variations, including food composition, storage temperature, packaging material characteristics, and duration of contact [45]. Of particular concern is the presence of cyano derivatives in cheese and noodles, as these compounds may degrade into cyanide, a substance known to interfere with thyroid and neurological functions [46]. The identification of nitrites in chocolates and chips is also noteworthy, given their potential to form carcinogenic nitrosamines under specific conditions [47].

Foods with high fat content, such as chips and dairy products, showed elevated migration levels, reinforcing previous findings that suggest lipids facilitate chemical absorption from packaging materials [48]. Moreover, the detection of unknown substances in noodle samples indicates the need for enhanced analytical techniques to determine their identity and possible effects on human health [49].

The findings underscore the importance of regulatory oversight and continuous monitoring to reduce consumer exposure to potentially harmful substances. Future research should focus on identifying unidentified compounds, assessing cumulative exposure risks, and developing safer alternatives for food contact materials.

**Figure 2:**
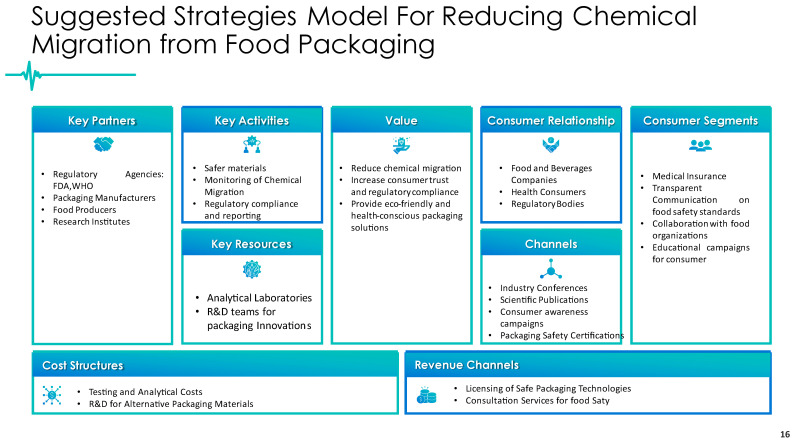
Suggested model for strategies to reduce chemical migration from food packaging [50,51].

The concept presented in Figure 2 was developed based on the findings of our study, complemented by relevant research on chemical migration prevention and regulatory frameworks [50,51]. The strategies outlined in the figure align with existing literature, governmental policies—including those established by the FDA, WHO, and EFSA—and recognized industry practices. Where applicable, references to scientific studies, regulatory reports, and policy documents will be provided to substantiate the model’s development.

The proposed framework aims to minimize chemical migration from food packaging while ensuring regulatory compliance and consumer protection. It emphasizes the role of key stakeholders, including regulatory agencies, packaging manufacturers, food producers, and research institutions, in fostering collaboration to develop safer packaging materials, implement effective monitoring strategies, and enforce compliance with safety standards. The model also highlights the importance of analytical laboratories and R&D teams as essential resources for innovation and scientific advancements in packaging safety.

This strategic approach is designed to reduce chemical migration risks, enhance consumer trust in food safety regulations, and promote the adoption of sustainable and health-conscious packaging solutions. The successful implementation of this framework requires active engagement with food companies, regulatory bodies, and consumers through scientific publications, industry conferences, and packaging safety certification programs.

Financial considerations within this strategy include the costs associated with analytical testing, regulatory compliance assessments, and R&D investments for alternative packaging materials. Potential revenue streams include technology licensing for safer packaging solutions and food safety consultation services.

Overall, this integrated model underscores the critical need for continuous monitoring, regulatory alignment, and the development of safer packaging alternatives to protect public health. The structured approach reflects both scientific evidence and regulatory priorities, ensuring that efforts to mitigate chemical migration risks are both effective and sustainable.

This study is limited by its geographical scope, which may not fully represent variations in packaging materials and regulatory standards across different countries. Furthermore, the detection of unidentified compounds highlights the need for advanced analytical techniques, such as high-resolution mass spectrometry and expanded reference databases, to improve compound identification and assess potential toxicological risks in future research.

## 5. Conclusions

This study aimed to investigate the migration of chemical compounds from plastic food packaging into various food categories and assess their potential health risks. The analysis identified nine chemical groups, including alanine, acetic acid, cyano derivatives, urea, amines, amides, benzene derivatives, nitrites, and non-specified compounds, with significant differences in migration levels across different food categories. Notably, high concentrations of benzene derivatives, nitrites, and amides were detected in chips, chocolates, and candies, raising concerns due to their potential carcinogenic and toxicological effects. These findings highlight the importance of continuous monitoring, advanced analytical techniques, and the development of safer packaging materials. Harmonizing international standards and expanding toxicological profiling of unknown compounds are essential to mitigate chemical migration risks and safeguard consumer health.

## Figures and Tables

**Table 1:** Sample group types, storage conditions, and storage periods.

Sample Type	No. of Samples	Storage Temperature °C	Storage Period
Frozen raw meat	16	−18 °C	48 h
Frozen raw chicken	14	−18 °C	48 h
Frozen Vegetables	25	−18 °C	48 h
Frozen fruits	18	−18 °C	48 h
Frozen sea food	18	−18 °C	48 h
Frozen fast food	35	−18 °C	48 h
Ice cream	17	−18 °C	48 h
Noodles	15	Room Temperature	72 h
Chips	15	Room Temperature	72 h
Chocolates	17	Room Temperature	72 h
Cheese Bars	15	5 °C	72 h
Total number of samples	205		
Total number of excluded samples	10		
Total numbers of analyzed samples	195		

**Table 2:** GC-MS parameters, sensitivity, and quality assurance.

Parameters	Specifications	
**GC Setting**		
Carrier Gas	Helium	Ultra-high-purity (UHP) helium (99.9999%)
Injector Temperature	120 °C	Optimizes sample volatilization
Oven Program	35 °C for 2 min, ramp at 10 °C/min to 200 °C, hold for 5 min	
Column	Capillary Column (30 m × 200 µm × 1.1 µm)	High-resolution separation for volatile compounds
**MS Setting**		
Ionoization Mode	Electron Impact (EI) at 70 eV	Fragmentation for compound identification
Mass Range	30–300 amu	
Oven Temperature Program	Initial 40 °C (held for 2 min), ramp to 250 °C at 10 °C/min	Optimized for separating compounds with a wide range of volatilities
Detector Type	Electron Ionization (EI)	Produces consistent fragmentation for accurate identification
Mass Range (*m*/*z*)	40–400	Covers the expected range of target compounds
Source Temperature	200 °C	
Full Scan Mode	0.41 s Scan Time, 0.01 s InterScan Delay	
PAHs Identification		Comparison with NIST Mass Spectral Library Confirms compound identity using reference spectra
Limit of Detection (LOD)	0.01 mg/kg	Minimum detectable concentration
Limit of Quantification (LOQ)	0.05 mg/kg	Minimum concentration for reliable quantification
Best Sensitivity Achieved	0.001 mg/kg	For acetic acid and nitrites Indicates highest detection capability

**Table 4:** Regression Analysis of Migration Levels Across Food Categories.

Chemical Compounds	Regression Coefficient (β)	*p*-Value	R^2^ Value	95% CI for β
Alanine	0.85	<0.01	0.72	[0.75–0.95]
Acetic Acid	0.78	<0.01	0.68	[0.70–0.86]
Cyano Derivatives	0.92	<0.001	0.81	[0.84–1.00]
Benzene Derivatives	0.88	<0.001	0.75	[0.78–0.98]
Amines	0.76	<0.01	0.65	[0.66–0.86]
Amides	0.84	<0.01	0.71	[0.74–0.94]
Nitrites	0.90	<0.001	0.78	[0.82–0.98]
Non-Specified Compounds	0.79	<0.01	0.69	[0.69–0.89]

## Data Availability

The data presented in this study are available on request from the corresponding author.
